# Chylous Cardiac Tamponade with Chylothoraces Secondary to Hodgkin's Lymphoma: Octreotide in Conjuncture with Standard of Care Dietary Fat Restriction

**DOI:** 10.1155/2019/1406840

**Published:** 2019-03-25

**Authors:** John O'Donnell, Jay Kirkham, Duane Monteith, Christopher Frontario, Rahul Sharma, Brianna Higgins

**Affiliations:** ^1^Rowan University School of Osteopathic Medicine, USA; ^2^Jefferson Health New Jersey – Department of Pulmonary/Critical Care, USA; ^3^Jefferson Health New Jersey – Department of Thoracic Surgery, USA; ^4^Children's Hospital of Philadelphia – Department of Dietetics and Nutrition, USA

## Abstract

Chylous effusions are a well-known complication from a variety of etiologies including trauma, malignancies, and anatomic defects, with the most common location being in the pleural space. A pericardial chylous effusion (chylopericardium) is uncommon, and a chylopericardium with concomitant bilateral chylous pleural effusions (chylothoraces) has only been reported in less than a handful of case reports. Our patient presented with bilateral chylothoraces and a chylopericardium with tamponade physiology secondary to Hodgkin's Lymphoma. In this article, we discuss our treatment of this patient with the somatostatin analogue octreotide, as well as the standard of care dietary fat restriction, in order to control these effusions until the patient's chemotherapy took effect.

## 1. Introduction

Chylous pericardial effusions are extremely rare but have been described in literature, originally noted to occur in 3% of patients with nontraumatic chylothoraces [1]. The only documented prevalence is 0.22% and 0.5% in pediatric cardiac operations, but this condition is so rare prevalence in adults has never been calculated and the presence of both bilateral chylothoraces and a chylopericardium has only been described in limited case studies [2]. In a literature review from 1996 to 2006, 33 cases of chylopericardium were identified in adults above 18 years of age with a confirmed diagnosis by laboratory testing and only 2 of these were the result of a lymphoid malignancy [3].

We report a case of a 39-year-old male recently diagnosed with Hodgkin's Lymphoma presenting to the hospital with dyspnea who was found to be in cardiac tamponade due to a chylopericardium with bilateral chylothoraces and a pericardial fluid triglyceride level of 135mg/dL and a known pleural triglyceride level of 775mg/dL from a sample take 10 days prior. He was taken to the operating room for an emergent pericardial window, right talc pleurodesis, and placement of a left tunneled pleural catheter. When the patient returned from the operating room, he was started on octreotide 100 mcg every 8 hours subcutaneously along with total parental nutrition. The patient was subsequently started on a <10g/day fat diet and by hospital day 5 the patient's fluid became serous and repeat fluid triglyceride level was measured at 46mg/dL. The patient's condition was significantly improved at this point and he was stable for discharge home to resume treatment for his lymphoma.

This case is unique as it is one of the first cases documented of chylopericardium with evidence of tamponade as well as bilateral chylous pleural effusions. This case is also the first case to use octreotide as well as a standard dietary fat restriction to treat a chylopericardium due to lymphoma. Octreotide has been documented in limited case reports and literary reviews as a potential therapy for treating chylothoraces, chylopericardium secondary to surgery, and chyloperitoneum [2–6]. Given this data, it was extrapolated that octreotide may provide benefit to patient's chylopericardium and chylothoraces from his Hodgkin's Lymphoma. This therapy resulted in the patient's pleural fluid triglyceride count to decrease by nearly 94% within 5 days of starting this therapy while maintaining a dietary fat restriction. With this case report, we hope to provide further evidence of a possible therapy to this markedly uncommon and potentially life-threatening complication.

## 2. Case Report

**Figure 1:**
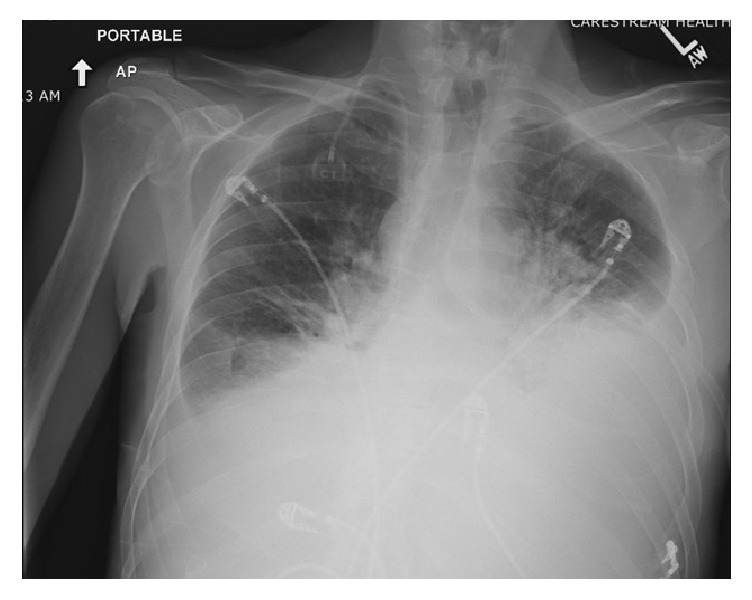
AP chest radiograph of the patient in the ED illustrating bilateral left greater than right chylous pleural effusions upon initial presentation.

**Figure 2:**
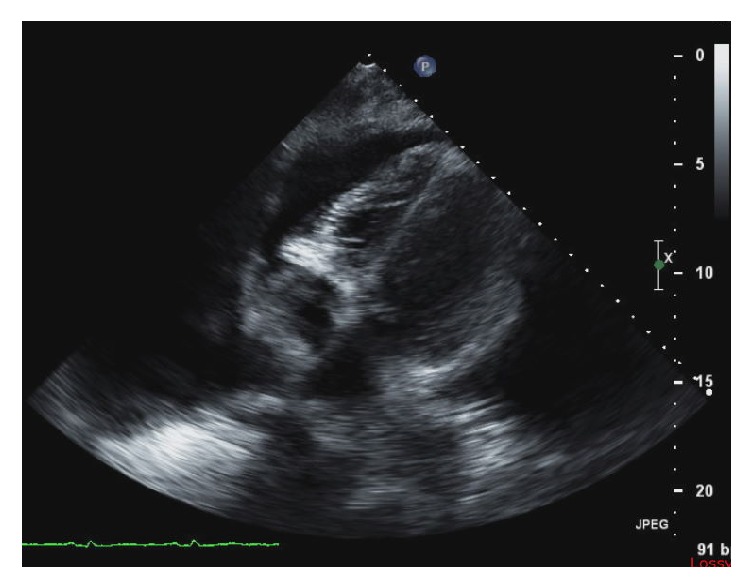
A transthoracic echocardiogram illustrating an apical four-chamber view of the patient's heart during diastole illustrating right ventricular free wall collapse.

A 39-year-old male with a past medical history of polysubstance abuse on methadone presented to the emergency department (ED) for worsening shortness of breath. He was recently diagnosed with Hodgkin's Lymphoma within the past month, after initially presenting with a left-sided chylous pleural effusion. He had required multiple thoracenteses over the past three weeks prior to admission to the hospital and was instructed to start a <20g/day fat diet due to the pleural fluid triglyceride level of 775mg/dL with 74% lymphocytes. The patient had started chemotherapy with Adriamycin, Adcetris, Vincristine, and Dacarbazine two days prior to presenting to the ED at his outpatient oncologist's office. In the ED, he was found to have an oxygen saturation of 88% on room air, which improved to 92% on 4L nasal cannula. The patient was frail appearing with temporal and diffuse muscle wasting with moderate respiratory distress. A chest radiograph was obtained, showing moderate bilateral pleural effusions that had reaccumulated over the past week since his last thoracentesis (Figure 1). The patient was evaluated by Cardiology while still in the ED and a STAT transthoracic echo was performed (Figure 2) showing compression of the right atrium and right ventricle during diastole. Cardiothoracic surgery was immediately consulted and the patient was taken for an emergent pericardial window.

**Figure 3:**
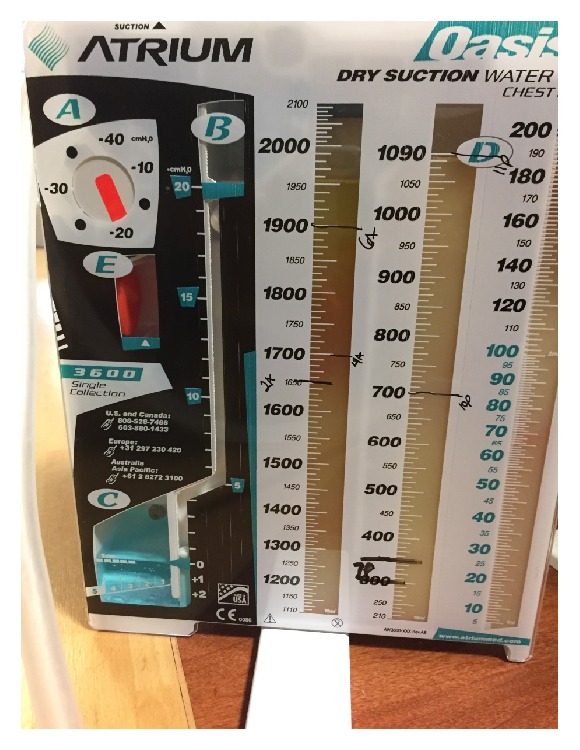
The patient's left chest tube with 1950cc of fluid drainage. You can clearly see the pleural fluid transitioning from pure chylous to almost completely serous.

A pericardial window was created with drainage to the right pleural space. Approximately 500mL of chylous fluid was drained from the pericardial space, 2L of similar fluid drained from the right pleural space, and 2.5L of chylous fluid was drained from the left pleural space through placement of a tunneled pleural catheter. Intraoperatively, pleural studding was noted throughout the right parietal pleura and pericardium. Biopsies were taken followed by talc pleurodesis. Pleural and pericardial fluid was sent for analysis, which showed a triglyceride level of 135mg/dL. The patient also had a serum triglyceride level of 105mg/dL. The patient was brought to the ICU and started on a fat-free, clear liquid diet and Octreotide 100mcg every 8 hours subcutaneously. Overnight to the morning of postoperative day (POD) 1, the patient drained another 1.9L of chylous fluid from the left chest tube (Figure 3) and 1L from the right chest tube. Both tubes were clamped to prevent further volume and protein loss. His diet was advanced to a regular <10g/day fat diet and a PICC line was placed to start total parenteral nutrition (TPN) in order to supplement nutrition with the total elimination of long chain fatty acids from the patient's diet. The patient had his chest tube clamped overnight with subsequent drainage every morning up to 1L from each side in order to limit further fluid and nutrition loss given his frail state. On POD 2, the patient was started on TPN and had 1.3L of chylous fluid drained from his left and 1L from his right chest tube. On POD 3, 1L of chylous fluid from the left and 900cc of chylous fluid was drained from the right. The patient was clinically stable and was downgraded from the ICU to telemetry where the chest tubes continued to be drained for 1L bilaterally and fluid color improved from milky white to serous. Repeat pleural fluid testing on POD 5 showed that the triglycerides had decreased to 46mg/dL and the patient's chest radiograph continued to improve. TPN was stopped and he tolerated 100% of his nutrition on an oral <10g/day fat diet. The patient's right chest tube was removed after output decreased to 250cc in 24 hours, and the output from the left tunneled pleural catheter continued to drain 1L daily of serous fluid. The patient's imaging remained stable and he was subsequently discharged home on POD 10 with thorough education on his dietary restrictions.

Unfortunately, due to lack of insurance, the patient could not continue the subcutaneous octreotide as he was unable to afford the out-of-pocket costs of the medication. Two days after discharge, he returned to the ED complaining of dyspnea. A chest radiograph at that time showed an unchanged small loculated pleural effusion on the right and a new large left pleural effusion. Only 1L of the estimated 2.5Ls in the pts left pleural space was drained from the patient's left tunneled pleural catheter which was milky white and was sent for analysis which showed a triglyceride level of 745mg/dL. He was reeducated on proper dietary habits and the importance of starting chemotherapy, which was initiated the next day. Two months later, the patient's repeat PET scan showed a significant decrease in the extent of the FDG avid foci consistent with the known lymphoma and small-to-moderate left pleural effusion which was draining <1L every 3 days at that time.

## 3. Discussion

Chylopericardium is an extremely rare finding that, in and of itself, has a high mortality rate. A very small subgroup of these patients present to the hospital in cardiac tamponade, a life-threatening emergency [7]. This disorder was first described in 1888 by Hasebrock; however, due to the low prevalence of this finding, there has been neither an established treatment regimen nor diagnostic criteria [3]. Staats et al. use the absolute triglyceride value >110mg/dL of the fluid to establish a diagnosis with >99% specificity [8], while Dib et al. demonstrate that ≥2 of 4 diagnostic criteria can be used with 100% sensitivity and specificity to establish a diagnosis (Table 1) [2]. Furthermore, in this study, none of the patients were treated with octreotide and literature searches reveal only limited cases, showing its use in the treatment of either chylothoraces, chylopericardium, or chyloperitoneum. None of these studies specifically utilized octreotide for the treatment of chylopericardium due to lymphoma. The only case of chylopericardium secondary to Hodgkin's Lymphoma was described by Raya et al. and was due to obstruction of the thoracic duct from an obstructive thymoma that converted to Hodgkin's Lymphoma [9].

Currently, the management of chylopericardium and more commonly chylothoraces is based on the etiology and consists of a strict dietary fat restriction, surgical intervention if indicated, and treating the underlying cause [4]. The potential causes of chylous effusions are extremely broad but primarily fall into 4 categories: malignancies, trauma, idiopathic, and miscellaneous with lymphoma being the most common nontraumatic cause [4, 10]. This variety of etiologies along with the very low prevalence in the nontraumatic adult population makes developing evidence based guidelines very challenging.

Octreotide has been used in limited case reports and reviews first noted in 1990 (as somatostatin) to treat chylothoraces. However, there is no set dosing regimen that has been shown to be more beneficial than another with dosages ranging from 0.3 to 40mcg/kg/hr in infants to 50-100mcg every 8 hours [6, 7, 11] in adults. Octreotide works by inhibiting gastrointestinal fluid secretion and triglyceride absorption, splanchnic blood flow, and thoracic duct flow which all decrease the accumulation of chyle [6, 7, 11]. This, as an adjuvant therapy to a low fat diet, has been shown in limited cases to decrease the size and output of effusions with response times ranging from 2 to 14 days, providing a decreased chyle production until the etiology can be effectively treated [6, 7, 11]. In this patient, the initiation of octreotide caused a significant decrease in the triglyceride count of the patient's pleural/pericardial fluid, while the patient was compliant. Talc pleurodesis has also been used to treat chylothoraces in patients with lymphoma with 100% documented success in previous studies [12–14]. This therapy worked well in our patient, which was observed when he returned with primarily a left-sided effusion and only minimal accumulation on the right side that had the pleurodesis.

This case is unique as it is one of the first cases documenting chylopericardium causing cardiac tamponade as well as bilateral chylous pleural effusions. This case is also the first case to use octreotide in addition to standard of care in order to treat a chylopericardium due to Hodgkin's Lymphoma. This therapy resulted in the patient's pleural fluid triglyceride count to decrease by nearly 94% within 5 days of starting this therapy, which is consistent with response times noted in previous studies of its use in treating chylous effusions. Subcutaneous octreotide in conjuncture with a <10g/day fat diet and talc pleurodesis resulted in significant improvement in the patient's symptomatology and quality of life providing adequate time for the patient's outpatient chemotherapy taking effect for his underlying Hodgkin's Lymphoma. With this case report, we hope to provide further evidence of a possible therapy to this markedly uncommon and potentially life-threatening complication.

## Figures and Tables

**Table 1:** Diagnostic criteria of chylous fluid analysis.

*Fluid appearance: milky yellow*	
(1) Triglyceride content	>500mg/dL
(2) Cholesterol/triglyceride ratio	<1.0
(3) Fluid cultures	Negative
(4) Fluid cell count	Lymphocytic predominance
